# A Modular Assembly Platform for Rapid Generation of DNA Constructs

**DOI:** 10.1038/srep16836

**Published:** 2016-02-18

**Authors:** Elliot H. Akama-Garren, Nikhil S. Joshi, Tuomas Tammela, Gregory P. Chang, Bethany L. Wagner, Da-Yae Lee, William M. Rideout III, Thales Papagiannakopoulos, Wen Xue, Tyler Jacks

**Affiliations:** 1 David H. Koch Institute for Integrative Cancer Research, Massachusetts Institute of Technology, Cambridge, MA 02139, USA; 2 Department of Biology, Massachusetts Institute of Technology, Cambridge, Massachusetts 02142, USA; 3 RNA Therapeutics Institute and Program in Molecular Medicine, University of Massachusetts Medical School, Worcester, MA 01605, USA; 4 Howard Hughes Medical Institute, Massachusetts Institute of Technology, Cambridge, Massachusetts 02139, USA

## Abstract

Traditional cloning methods have limitations on the number of DNA fragments that can be simultaneously manipulated, which dramatically slows the pace of molecular assembly. Here we describe GMAP, a Gibson assembly-based modular assembly platform consisting of a collection of promoters and genes, which allows for one-step production of DNA constructs. GMAP facilitates rapid assembly of expression and viral constructs using modular genetic components, as well as increasingly complicated genetic tools using contextually relevant genomic elements. Our data demonstrate the applicability of GMAP toward the validation of synthetic promoters, identification of potent RNAi constructs, establishment of inducible lentiviral systems, tumor initiation in genetically engineered mouse models, and gene-targeting for the generation of knock-in mice. GMAP represents a recombinant DNA technology designed for widespread circulation and easy adaptation for other uses, such as synthetic biology, genetic screens, and CRISPR-Cas9.

For decades, molecular cloning has allowed for manipulation of recombinant DNA to assemble DNA constructs that are widely used in molecular and synthetic biology. Most commonly, approaches to join DNA molecules take advantage of the specificity of restriction endonucleases and PCR to create compatible ends that can be joined using DNA ligase^1^. These traditional cloning methods rely on the presence of restriction sites in both vector and insert, and their prevalence – or lack thereof – can constrain possible assemblies, in particular those involving multiple inserts.

Several cloning methods have been developed to overcome these constraints, therefore allowing for high-throughput assembly of DNA constructs. Gateway Cloning and other similar site-specific recombination platforms, such as Creator, Echo, and Univector, rely on recombinase to recombine inserts between vectors. However, these methods are limited to “destination” vectors with appropriate recombination sequences and only allow cloning of one insert at a time. Moreover, they require proprietary enzyme mixes and are expensive relative to traditional cloning methods. New cloning strategies developed within the past decade, such as sequence and ligation-independent cloning^2^,^3^, Golden Gate Assembly^4^,^5^,^6^, and Gibson Assembly^7^,^8^, overcome these sequence requirements and allow for assembly of multiple inserts in a given reaction, particularly toward the engineering and study of synthetic biology pathways.

Recently, several frameworks to facilitate modular assembly of DNA constructs have been developed. In order to assemble large genetic circuits of multiple transcriptional units, Guye *et al.* designed a set of unique nucleotide sequences that when combined with Gateway Cloning and Gibson Assembly facilitate construction of a single construct used for transfection and stable integration in human cells^9^. A similar approach involves preparation of part libraries via BioBrick Assembly, which are then digested and assembled using Gibson Assembly into destination vectors for bacterial expression or genomic integration into mammalian stem cells^10^. Modular Overlap-Directed Assembly with Linkers (MODAL) relies on a software tool to design overlap sequences for Gibson Assembly into yeast and bacterial plasmids^11^. Several Golden Gate based platforms have also been developed for modular scar-benign assembly of plasmids for *in planta* transformation^12^,^13^. With these previous advances in synthetic biology circuit design in mind, we sought to develop a modular assembly platform using libraries of promoters, genes, and destination vectors applicable towards a broader range of techniques common to biomedical research, such as viral production and gene targeting.

Here we introduce GMAP (Gibson assembly-based modular assembly platform), which uses Gibson Assembly to facilitate the modular assembly of DNA constructs from established collections of promoters, genes, and backbones. GMAP reduces the time frame from the conception of an idea (gene knockdown, overexpression, conditional expression) to construct design, assembly, and screening to less than three days (Supplementary Fig. S1) and is easily adapted to any destination construct of interest. In particular, we establish a common platform for assembly of genetic tools, ranging from expression and viral constructs to homologous recombination targeting constructs, in order to address questions of increasing biologic complexity.

As the basis for GMAP, we adopted the Gibson assembly method^7^, which utilizes 30–40 base pair (bp) “overlap” sequences between two DNA segments to generate a fusion product (Fig. 1a). Traditionally, overlap sequences are fragment-specific and create seamless assembly of fragments. However, in order to make GMAP modular, we designed five common overlap sequences (sites #1–5) to designate specified positions within the construct (Fig. 1b, Supplementary Fig. S1). These five sequences were used to establish collections of over 30 promoters and 140 genes (Supplementary Table S1) using the PCR scheme outlined in Supplementary Figure S2, and that continue to expand with ongoing experiments. This PCR scheme forms the basis of a four fragment design that consists of promoter A (pA):gene A (gA) – promoter B (pB):gene B (gB) or a two fragment design (pC:gB or pA:gC), therefore allowing for modular, parts-based assembly of DNA constructs (Fig. 1b). As isothermal assembly and *E. coli* transformation require 30 min, parts can be generated and assembled within a single day.

Each 30 bp overlap sequence encodes a unique restriction enzyme site to facilitate rapid screening (Fig. 1b). We also designed a series of six sequencing primers that anneal to each 30 bp overlap (Supplementary Table S2), allowing for screening via Sanger sequencing. In addition, we optimized reaction parameters and suggest using 200 ng of backbone and incubating for at least 20 min for maximal efficiency of assembly of the desired construct (Supplementary Fig. S1). As reaction efficiency decreases with increasing fragment number (Supplementary Fig. S1), one must screen at least three bacterial colonies for two-fragment (not including backbone) reactions and five for four-fragment reactions to have a greater than 99% probability of obtaining at least one correctly assembled colony.

In order to demonstrate the feasibility of using GMAP to create widely applicable expression constructs, we first created lentiviral and retroviral constructs (Supplementary Fig. S3). A GMAP-compatible pLL3 lentiviral backbone was designed by replacing all components between the Psi element and the WPRE sequence with a DNA gene block (Supplementary Table S2) such that linearization with *PmeI* and *BsrGI* yields a lentiviral backbone with the #1 and #5 sites at the 3′ and 5′ ends, respectively. Similarly, in order to generate a GMAP-compatible MSCV retroviral backbone, a DNA gene block (Supplementary Table S2) replaced all elements between the Psi element and viral long terminal repeat (LTR) such that linearization with *PmeI* yields sites #2 and #5 at the 3′ and 5′ ends, respectively. This process is easily adapted to other destination constructs of interest by cloning the appropriate GMAP cassette from Supplementary Table S2 into the desired plasmid backbone (see Methods).

We used these GMAP-compatible viral backbones to simultaneously assemble series of retroviral and lentiviral constructs with genes and promoters typically used in biomedical research. Using ubiquitous and tissue-specific promoters of different strengths, we assembled a series of six retroviral constructs with a unique promoter driving expression of GFP. We compared the relative strengths of these promoters by transducing a murine lung cancer (KP) cell line or murine 3T6 fibroblast cells with each retrovirus and selecting with puromycin (Fig. 1c). GFP expression was then analyzed by flow cytometry, demonstrating that CMV in lung cancer cells and CCSP in 3T6 cells were the strongest and weakest promoters respectively, corresponding to a 54.3 ± 5.33-fold difference in median fluorescence intensity (MFI, Fig. 1d).

Another common application of DNA constructs is RNA interference experiments, which are particularly amenable to the modularity and rapidity of GMAP. As such, we used GMAP to assemble a series of three lentiviral “sensor” constructs^14^, each with a unique fluorescent protein that contains a short hairpin RNA (shRNA) target sequence in its 3′UTR (Supplementary Fig. S4). We produced such lentiviral constructs with mTagBFP2-A^UTR^, mKate2-B^UTR^, or mKusabira-Orange(KO2)-C^UTR^ in the gB position and used them to transduce 3TB cells. We developed 3TB cells, a 3T6-derived reporter line engineered to become blasticidin resistant after exposure to Cre recombinase (Cre), for easy *in vitro* validation of Cre-expressing constructs (Supplementary Fig. S5). After transduction, sensor 3TB cells were selected with hygromycin to establish cell lines stably expressing fluorescent reporters sensitive to shRNA-mediated knockdown (Fig. 1e). In order to identify effective shRNAs, we used GMAP to assemble three lentiviral constructs containing doxycycline-inducible hairpins, the reverse tetracycline-transactivator (rtTA3), and Cre. We then transfected each sensor 3TB line with an inducible shRNA construct and selected with blasticidin (Supplementary Fig. S6). Upon treatment with doxycycline, cells inducibly expressed shRNA and knockdown of the endogenous fluorescent protein was assessed by flow cytometry, identifying shRNA “C” as the most potent shRNA (Fig. 1f–h). These results demonstrate the ability of GMAP to rapidly assemble and functionally test *in vitro* retroviral and lentiviral constructs using standard genetic components such as synthetic promoters, tetracycline response elements, and shRNAs.

The simplicity and speed of GMAP allows one to assemble not only prototypical expression constructs, but also constructs of increasing genetic complexity and biological relevance. In order to demonstrate the benefit of GMAP’s modularity and non-reliance on unique restriction sites, we used GMAP to assess hypoxia in a genetically engineered mouse model of non-small cell lung cancer (NSCLC) using an advanced hypoxia response element (HRE):GFP reporter cassette^15^. Using GMAP we generated a HRE:GFP-pGK:Cre lentivirus, which was delivered intratracheally to *K-ras*^*LSL−G12D/+*^*;p53*^*fl/fl*^ mice^16^, resulting in the development of lung adenocarcinoma. Thirty weeks after tumor induction mice were injected with pimonidazole prior to sacrifice, which allowed us to objectively visualize areas of hypoxia using immunohistochemistry for pimonidazole adducts that form in hypoxic regions of the tumor. Immunohistochemistry revealed that GFP expression co-localized with areas of pimonidazole staining (Fig. 2a), demonstrating the accuracy of the HRE:GFP reporter lentivirus and its potential application to visualize intratumoral hypoxia, in concordance with previously published data using tumor xenografts^17^,^18^,^19^. These data demonstrate just one of many potential *in vivo* applications of GMAP, which allows for easy assembly of lentiviral constructs expressing biologically relevant sequences of unlimited complexity. For example, the modular ability of GMAP enables high throughput production of constructs expressing components of the clustered regularly interspaced short palindromic repeats (CRISPR)-Cas9 system^20^, providing a simple method to study genome editing and to perform genetic screens^21^,^22^.

Beyond biologically relevant expression constructs, GMAP facilitates high-throughput creation of novel genetic tools with greater ease and speed than traditional cloning methods. One such tool is homologous recombination constructions designed to mediate targeted genome modification and generate knock-in animals^23^,^24^. In order to facilitate rapid generation of knock-in animals, we created a GMAP-compatible backbone containing Rosa26 homology arms (R26TV), designed to target a CAG-driven loxP-stop-loxP (LSL) cassette into the *Rosa26* locus (Supplementary Fig. S3), such that sequences inserted via GMAP are expressed following exposure to Cre. Using GMAP, we assembled a R26TV LSL construct driving expression of the tetracycline transrepressor (tTR) from *Escherichia coli Tn10* fused to the KRAB domain of human Kox1 (tTR-KRAB) linked via a P2A peptide to rtTA3 linked via a degenerate P2A peptide to luciferase. We first validated the tight regulation of tetracycline-response elements by tTR-KRAB-rtTA3-Luc (TRL) *in vitro* by generating a KP-derived cell line engineered to inducibly express CloverCP (VerdeGo). VerdeGo cells were transduced using a GMAP-generated lentivirus encoding the full TRL sequence and shown to express CloverCP in a doxycycline-dependent manner (Fig. 2c,d). Following *in vitro* validation, C57BL/6J*-Tyr*^* c−2J*^ embryonic stem (ES) cells were electroporated with R26TV-LSL-TRL and five clones were generated, one of which was positive for *Rosa26* integration by PCR screening and Southern analysis (Fig. 2f, Supplementary Fig. S7). These targeted ES cells report Cre recombination via both luciferase (Fig. 2g, Supplementary Fig. S7) and doxycycline-inducible expression when combined with a pTRE:iRFP670-EFS:Cre-2A-GFP lentivirus generated by GMAP (Fig. 2h, Supplementary Fig. S7). These results demonstrate the versatility of GMAP to create genetic tools of increasing complexity, in particular toward the rapid validation and generation of knock-in mice.

GMAP provides a platform to address questions of increasing biologic complexity using genetic tools, ranging from commonplace expression constructs using simple synthetic elements to homologous recombination constructs using complex genomic sequences. Additionally, the establishment and continuous expansion of a GMAP-compatible collection of promoters, genes, and backbones provides a system for the rapid translation of an idea from *in vitro* testing using retrovirus, to *in vivo* testing using lentivirus, and finally to generation of knock-in mice using Rosa26 homologous recombination targeting constructs. We have designed GMAP to be easily and inexpensively adaptable for the creation of compatible backbones as well as promoter and gene collections. Recent advances in the fields of synthetic biology and genetics, particularly the widespread use of CRISPR-Cas9^25^, rely on the ease of construction of increasingly more complicated DNA constructs. As genomics and systems biology lead to the identification of novel genes and pathways of interest, efficient assembly of DNA constructs to interrogate these genes and pathways will become increasingly important. Together with its adaptability for various applications and for use by other investigators, GMAP provides a modular assembly platform that simplifies and accelerates this discovery process.

## Methods

### Oligonucleotide design

We designed five 30 bp overlap sequences to flank our four possible fragments in each construct. The sequences are: site #1, GATCAGTGTGAGGGAGTGTAAAGCTGGTTT; site #2, CTAACTCGAACGCTAGCTGTGCGATCGTTT; site #3, AAACCGCTGTTCCTAGGAATCCCGAGGCCT; site #4, GACCCGACATTAGCGCTACAGCTTAAGCGG; site #5, AAACGTTGTTGTTTGGGGTTGAATTACTCT. In order to amplify compatible genes and promoters, primers were designed to include the appropriate sites as primer overhangs in addition to standard PCR homology sequences. Primers were purchased from Integrated DNA Technologies (IDT) unmodified with standard desalting; sequences are available in Supplementary Table S3.

### Preparation of promoter and gene collections

**Figure 1:**
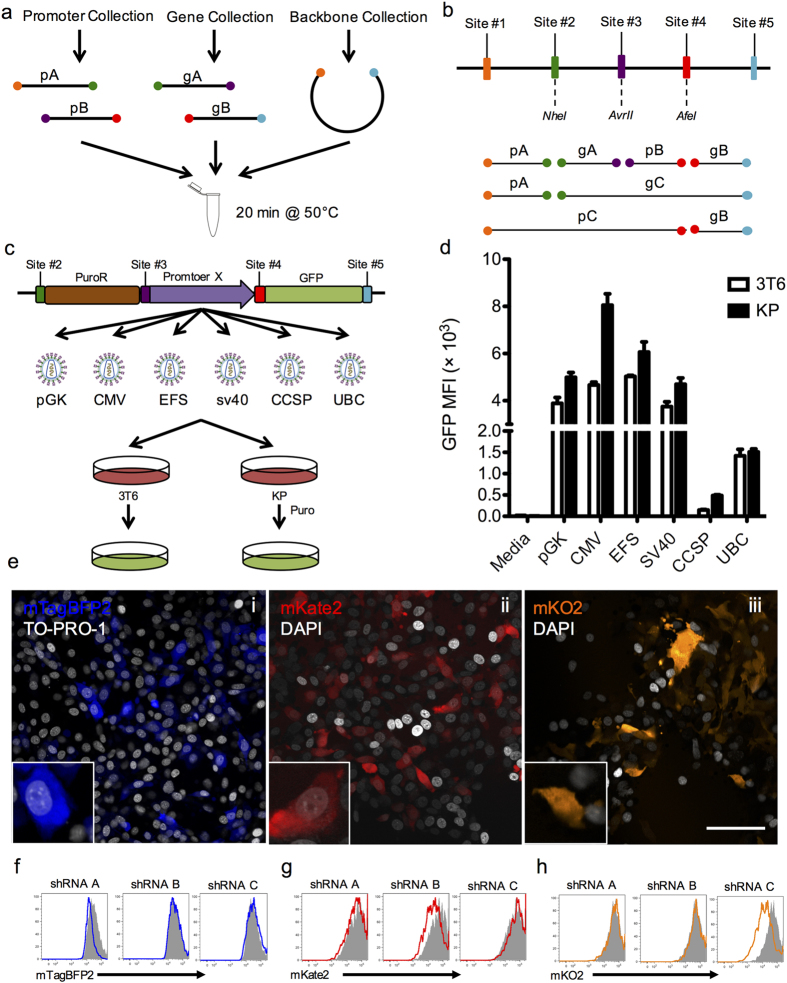
Gibson assembly-based modular assembly platform (GMAP). (**A**) Promoters, genes, and backbones from established GMAP-compatible collections are used in a one step isothermal assembly reaction to produce DNA constructs on demand. (**B**) Schematic of possible orderings of genes and promoters. Promoter A (pA) is flanked by sites 1 and 2, promoter B (pB) is flanked by sites 3 and 4, promoter C (pC) is flanked by sites 1 and 3, gene A (gA) is flanked by sites 2 and 3, gene B (gB) is flanked by sites 4 and 5, and gene C (gC) is flanked by sites 2 and 5. (**C**) Schematic diagram of experiment using GMAP retroviral backbone. Retroviruses expressing GFP driven by different promoters were assembled using GMAP and used to transduce murine 3T6 fibroblasts or murine lung cancer (KP) cells. PuroR, puromycin resistance; pGK, human phosphoglycerate kinase 1 promoter; CMV, cytomegalovirus immediate-early promoter; EFS, elongation factor 1α promoter; SV40, simian virus early 40 promoter; CCSP, clara cell secretory protein promoter; UBC, human Ubiquitin C promoter. (**D**) Bar graph shows flow cytometry measurements of median fluorescence intensity (MFI) of GFP from 3T6 and KP cells transduced with GMAP-generated retrovirus. Data are representative of at least three independent experiments. (**E**) GMAP-generated lentiviruses expressing mTagBFP2-A^UTR^ (i), mKate2-B^UTR^ (ii), or mKO2-C^UTR^(iii) sensor cassettes were assembled and used to transduce a Cre reporter cell line (3TB). 3TB cells were selected with hygromycin and visualized by confocal microscopy. Insets show higher magnification. Scale bar, 100 μm. (**F-H**) Histograms from 3TB cells expressing mTagBFP2-A^UTR^ (**F**), mKate2-B^UTR^ (**G**), or mKO2-C^UTR^ (**H**) transfected with three inducible hairpin constructs targeting the A, B, or C 3’UTRs assembled using GMAP. After transfection 3TB cells were selected with blasticidin, treated with doxycycline, and knockdown was assessed by flow cytometry analysis on GFP^+^ cells. Grey histograms represent cells lines transfected with an inducible shRNA targeting luciferase. Data are representative of at least three independent experiments.

Promoters were named pA, pB, or pC and genes were named gA, gB, or gC to designate their location in the four fragment scheme (Fig. 1b). To produce pA, F1 and R2 primers are used; to produce pB, F3 and R4 primers are used; to produce pC, F1 and R4 primers are used. To produce gA, F2 and R3 primers are used; to produce gB, F4 and R5 primers are used; to produce gC, F2 and R5 primers are used. Promoters and genes were amplified using PrimeSTAR HS (Takara) with an annealing temperature of 62 °C and extension lengths of 1 min/kb. PCR products were extracted from agarose gels after electrophoresis using the QIAquick Gel Extraction kit (Qiagen) or purified using the QIAquick PCR Purification kit (Qiagen) according to the manufacturer’s protocol. Promoters and genes were then adjusted to 57 nM with Tris-EDTA buffer (pH = 8.0), entered into the online database (Supplementary Table S1), and stored at −20 °C. We have generated GMAP-compatible TOPO constructs, which may serve as templates for investigators to amplify their own GMAP promoter and gene collections using primers from Supplementary Table S3. All TOPO constructs containing the promoters and genes described herein have been deposited in Addgene.

### Preparation of backbones

Compatible backbones were created by designing gene blocks (gBlocks) from IDT (Supplementary Table S2) to clone into viral or R26TV constructs such that digestion and gel purification would yield linearized backbones with sites #1 and 5 or sites #2 and 5 terminal. The retroviral backbone (RV 2-5) was created by cloning “RV 2–5 gBlock” into MSCV linearized with *BglII* and *ClaI* using Gibson Assembly such that digestion with *PmeI* followed by PCR purification yields a GMAP compatible backbone. The lentiviral backbone (LV 1–5) was created by cloning “LV 1–5 gBlock” into pLL3 linearized with *XmaI* and *AscI* using Gibson Assembly such that digestion with *PmeI* and *BsrGI* eliminates the 469 bp spacer sequence between sites #1 and 5. The CAG-driven R26TV LSL backbone (R26TV CAG LSL 2−5) was created by cloning “Rosa26 LSL 2–5 gBlock” into a R26TV LSL-GFP plasmid (Addgene plasmid 16103) linearized with *Asc* and *XmaI* such that digestion with *PmeI* eliminates a 389 bp spacer sequence between sites #2 and 5. This targeting construct has 5′ and 3′ homologous arms of 1.1 and 4.3 kb, respectively. TOPO backbones were created by linearizing PCR-BluntII TOPO with *BamHI* and cloning in “TOPO 1-4 gBlock” or “TOPO 2-5 gBlock” using Gibson Assembly such that digestion with *PmeI* and *NheI* eliminates a 361 bp spacer sequence between sites #1 and 4, or digestion with *PmeI* eliminates a 389 bp spacer sequence between sites #2 and 5, respectively. Following gel extraction, linearized backbones were adjusted to 57nM with Tris-EDTA buffer (pH = 8.0). All GMAP backbones described herein have been deposited in Addgene.

### One-step isothermal assembly

DNA constructs were assembled from our collections of promoters, genes, and backbones (Supplementary Table S1) using Gibson Assembly^7^. Briefly, 5X isothermal assembly reaction buffer was prepared by combining 3 mL of 1 M Tris-HCl (pH = 7.5), 300 μL of 1 M MgCl_2_, 600 μL of 10 mM dNTPs, 300 μL of 1M DTT, 1.5 g of PEG-8000, 20 mg of NAD, and water up to 6 mL, aliquoted and stored at −20 °C. Isothermal master mix was prepared by combining 320 μL of 5X isothermal assembly reaction buffer, 1.2 μL of T5 exonuclease (NEB), 20 μL of Phusion polymerase (NEB), 160 μL of Taq ligase (NEB), and 700 μL of water, aliquoted and stored at −20 °C. For each individual reaction, 15 μL of isothermal master mix was added to 5 μL of promoter, genes, and inserts (5.7 × 10^−2^ pmol each) and incubated at 50 °C for 20 min (Supplementary Figure S1). This reaction mix was then transformed into competent bacteria, and screened using *XmaI, NheI, AvrII, AfeI,* or *AscI*.

### Establishment of 3TB cell line

The sequence for blasticidin resistance was PCR amplified and cloned using Gibson Assembly into a pcDNA5 donor vector such that it is inverted and flanked by two sets of incompatible loxP sequences. The inverted and floxed blasticidin resistance sequence was then PCR amplified and cloned using Gibson Assembly into a MSCV pGK-PuromycinR vector such that inverted blasticidin resistance expression is driven by the retroviral LTR (FFiBlast MSCV Puro). Murine 3T6 fibroblasts were transduced using FFiBlast MSCV Puro, selected in 5 μg/mL puromycin (Life Technologies), single cell cloned, and screened using CMV-Cre adenovirus (Ad-Cre). A Cre-responsive clone was expanded for *in vitro* use (3TB).

### Cell culture

Human embryonic kidney cells (HEK-293FS), 3TB cells, and KP cells were cultured in DMEM (Corning) supplemented with L-glutamine (2 mM), penicillin (100 U/mL), streptomyocin (100 μg/mL), and 10% fetal calf serum (FCS; Gibco) at 37 °C in a 5% CO_2_ humidified atmosphere. Embryonic stem (ES) cells were cultured on primary irradiated mouse embryonic fibroblasts in Knockout DMEM (Life Technologies) supplemented with β-mercaptoethanol (100 μM), L-glutamine (2 mM), penicillin (100 U/mL), streptomycin (100 μg/mL), 1X Non-essential Amino Acids (Sigma), leukemia inhibitory factor (200 pg/mL; Amsbio), CHIR99021 (3 μM; P212121) and PD0325901 (1 μM; Selleck Chemicals), and 15% FCS at 37° in a 5% CO_2_ humidified atmosphere. Following transduction of 3TB or KP cells with lentivirus or retrovirus, 5 μg/mL puromycin (Life Technologies), 400 μg/mL hygromycin (EMD Millipore), 400 μg/mL zeocin (Life Technologies), or 20 μg/mL blasticidin (Invitrogen) selection was applied, as appropriate. To induce shRNA expression, transfected 3TB cells were treated with 5 μg/mL doxycycline (Sigma) for 48 hr. To induce CloverCP expression, VerdeGo cells were treated with 1 μg/mL doxycycline for 7 days. To induce iRFP670 expression, ES cells were treated with varying doxycycline for 3 days.

### Viral production and transduction

For transfection experiments, HEK-293FS cells were transfected using *Trans*IT-LT1 (Mirus Bio) in Opti-MEM (Life Technologies) and 3TB cells were transfected using Attractene Transfection Reagent (Qiagen) in Opti-MEM. Retrovirus was produced by transfection of 5 × 10^5^ HEK-293FS cells with 1 μg Phoenix-E and 2μg retroviral construct and supernatant filtration through 0.45 μm syringe filters (VWR). Lentivirus was produced by transfection of HEK-293FS cells with Δ8.2 (gag/pol), CMV-VSV-G, and lentiviral construct as previously described^16^.

### Rosa26 homologous recombination

For targeting experiments, 40 μg of R26TV was linearized with *AsiSI* (NEB) and electroporated into 1 × 10^6^ C57BL/6J*-Tyr *^*c−2J*^ ES cells with a single pulse of 600V, 25 μF. After 24 hr cells were selected with 300 μg/mL G418 (Life Technologies) for 7 days, followed by isolation of five subclones.

### Confocal microscopy

Cells were plated on Cover Glass Circles (Fisher) at 250 cells/mm^2^. After 24 h cells were washed with PBS and nuclear staining was accomplished using DAPI (5 μg/mL) or TO-PRO-1 (Invitrogen) prior to fixation with 1% paraformaldehyde (PFA, Electron Microscopy Sciences) and mounting with Vectashield Mounting Medium (Vector Laboratories). Images were acquired on an Olympus FV1200 Laser Scanning Confocal Microscope and analyzed with ImageJ (NIH, Bethesda, MD).

### Flow cytometry

Flow cytometry data were collected after 3–7 days of selection. Cells were trypsinized and fixed with Cytofix/Cytoperm (BD) and read on a BD LSR II HTS-2. Data was analyzed using Flowjo software (Tree Star).

### Mice

All animal studies described in this study were performed in accordance with protocols approved by the MIT Institutional Animal Care and Use Committee. All animals were maintained on a mixed C57BL/6J × 129SvJ genetic background. *K-ras*^*LSL−G12D/+*^*; p53*^*fl/fl*^ mice have been previously described^26^,^27^. Mice were infected intratracheally with 1 × 10^4^ transforming units lentivirus as described^16^ and examined after 30 weeks.

### Immunohistochemistry

To visualize hypoxic areas by immunohistochemistry, a commercially available hypoxyprobe kit (Hypoxyprobe™-1 Omni) was utilized. Pimonidazole hydrochloride was injected intraperitoneally into tumor-bearing mice at a dose of 60 mg/kg body weight 1 h before euthanasia. Lungs were perfused through the trachea with 4% PFA, fixed overnight, transferred to 70% ethanol and subsequently embedded in paraffin. Sections were cut at a thickness of 4 μm and stained with hematoxylin and eosin for pathological examination. Slides were antigen retrieved using Thermo citrate buffer, pH 6.0 and treated with Peroxidase and Alkaline Phosphatase Block (Dako), normal horse serum (Vector Labs), primary antibody, and anti-rabbit (Vector Labs) HRP-polymer. The slides were developed with ImmPACT DAB Peroxidase (Vector Labs), counterstained with haematoxylin in a Thermo Gemini stainer and coverslips added using the Thermo Consul cover slipper. The following antibodies were used for IHC: anti-TTF1 / Nkx2.1 (Epitomics, EP1584Y, 1:1,200), anti-GFP (Cell Signaling, 2956, 1:100), and anti-Pimonidazole (Hypoxyprobe, 1:500). All images were obtained using a Nikon 80i microscope with a DS-U3 camera and NIS-elements software.

### Southern analysis

Genomic DNA was isolated from ES cell subclones as previously described^28^ and digested with *BamHI* (NEB) overnight. Digestions were electrophoresed on 0.7% agarose gels and blotted to Amersham Hybond-N+ nylon membranes (GE Healthcare). ^32^P-labeled 5′ probe was synthesized using BW13 (AGACAAAACCCAGAGCCCAGAGC) and BW14 (TTGGGCCTAACTCGAGTCTCGCT) and applied in Church buffer^29^.

### PCR analysis

Primers to detect the *R26LSL-TRL* allele (R26For, GAAGAGGCTGTGCTTTGGG; R26Rev, ACCACTGGAAAGACCGCGAAGAG) were designed by the Primer3 program (www.http://biotools.umassmed.edu/bioapps/primer3_www.cgi). PCR amplifications were performed on 50 ng of genomic DNA using Green *Taq* DNA Polymerase (GeneScript). Targeted *Rosa26* allele yields a 1302 bp product and wild type *Rosa26* yields no product.

### Luciferase analysis

ES cells (1 × 10^4^) were seed in each well of a 48-well and transduced with varying Ad-Cre. After 3 days, 150 μg/mL D-Luciferin (Perkin Elmer) was applied and cells were imaged using the IVIS Spectrum Imaging System (Perkin Elmer). Cells were then washed with PBS, lysed with 30 μL Cell Culture Lysis Reagent (Promega), and supernatant was incubated with Luciferase Assay Reagent (Promega). Luminescence was measured using a Tecan Infinite M200 PRO Plate Reader.

### Crystal violet stain

Transduced 3T6 fibroblast subclones (2 × 10^4^) were seed in a 24-well and transduced with Ad-Cre (MOI=100). After 48 h, 20 μg/mL blasticidin was applied and after 4 days cells were washed with PBS and fixed with 1% PFA. Cells were then washed with PBS, stained with 0.5% crystal violet in 2.5% methanol for 30 min, and rinsed in dH_2_O.

### Statistical analysis

Statistical analysis was performed using unpaired two-tailed Student *t* tests in Prism 5 (Graphpad Software). Data are presented as mean ± standard error of the mean.

## Additional Information

**Accession Codes**: The following plasmids were deposited into Addgene: TOPO 2-5, TOPO 1-4, RV 2-5, LV 1-5, R26TV CAG LSL 2-5, TOPO mTagBFP2, TOPO mKate2, TOPO mKO2, TOPO iRFP670, TOPO CloverCP, TOPO HygroR, TOPO ZeoR, TOPO PuroR, TOPO rtTA3-2A-Cre, TOPO tTRKRAB- rtTA3-Luc, TOPO Cre-2A-GFP, TOPO EFS, TOPO pGK, TOPO CMV, TOPO CCSP, TOPO sv40, TOPO UBC, TOPO pTRE, and TOPO HRE. 3TB cells and VerdeGo cells are available upon request.

**How to cite this article**: Akama-Garren, E. H. *et al.* A Modular Assembly Platform for Rapid Generation of DNA Constructs. *Sci. Rep.* **6**, 16836; doi: 10.1038/srep16836 (2016).

## Supplementary Material

Supplementary Information

Supplementary Tables

## Figures and Tables

**Figure 2:**
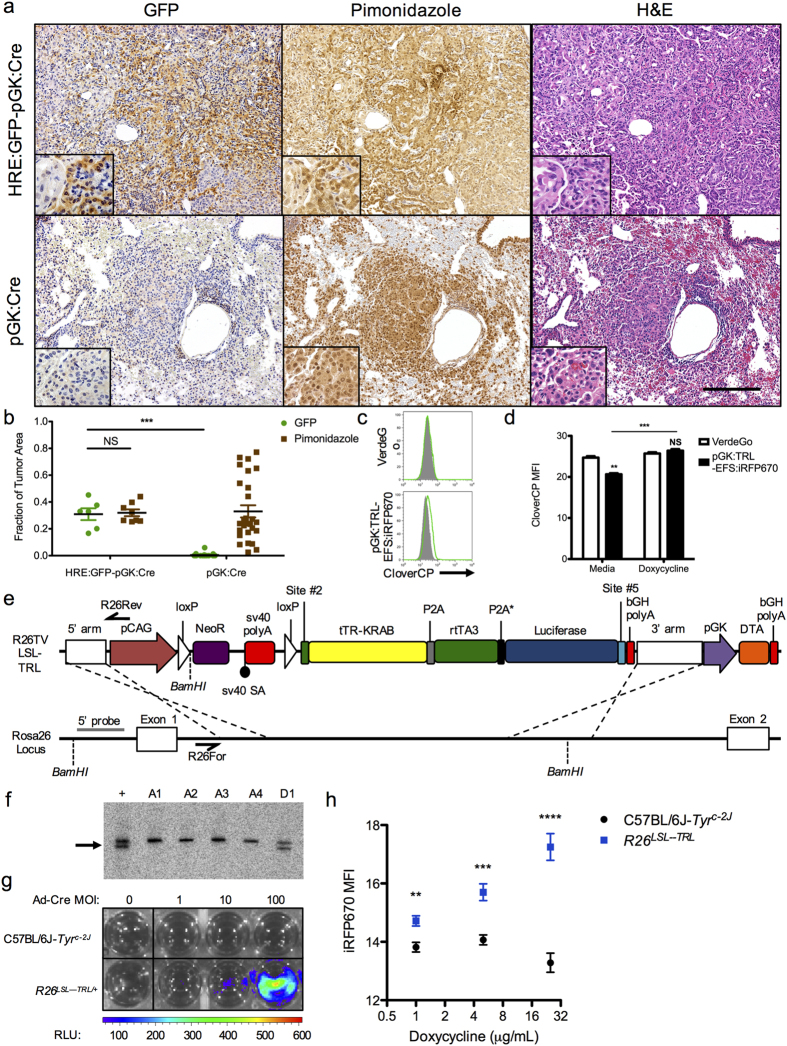
*In vivo* applications of GMAP using lentiviral and Rosa26 targeting constructs. (**A**) *K-ras*^*LSL−G12D/+*^*;p53*^*fl/fl*^ mice were infected with GMAP-generated hypoxia response element (HRE):GFP-pGK:Cre or pGK:Cre lentivirus. After 30 weeks tumors were assessed for GFP expression, hypoxia as determined by pimonidazole staining, and hematoxylin and eosin (H&E). Insets show higher magnification. Data are representative of at least three independent experiments. Scale bar, 200 μm. (**B**) Column scatter plot shows quantification of immunohistochemistry shown in (**A**). Data are presented as fraction of tumor area that expressed GFP or pimonidazole staining. NS, p > 0.05; ***p = 5.82 × 10^−12^. (**C**) FACS histogram of KP cells engineered to express CloverCP in the presence of doxycycline and rtTA (VerdeGo) that were transduced with a GMAP-generated pGK:tTR-KRAB-rtTA3-Luc (TRL)-EFS:iRFP670 lentivirus and treated with media (grey histogram) or doxycycline (green line). Data are representative of at least three independent replicates. (**D**) Bar graph shows quantification of flow cytometry shown in (**C**). NS, p > 0.05; **p = 7.68 × 10^−4^; ***p = 2.46 × 10^−4^. (**E**) Map of a GMAP-generated Rosa26 targeting vector (R26TV) and scheme for knock-in of a Cre-dependent tTR-KRAB-rtTA3-Luc construct into the *Rosa26* locus. LSL, loxP-Stop-loxP; NeoR, neomycin resistance; bGH polyA, bovine growth hormone polyadenylation signal; DTA, diphtheria toxin fragment A. Relevant primer binding sites (black half arrow) for PCR screening and restriction sites and probe (grey line) for Southern analysis are shown. (**F**) Southern analysis of ES cell DNA. C57BL/6J*-Tyr*^*c-2J*^ ES cells were electroporated with *AsiSI*-linearized R26TV and subclone genomic DNA was digested with *BamHI* and probed, yielding a 5.8 kb product for the wild type *Rosa26* locus and a 4.8 kb product for the targeted *Rosa26* allele. A1-D1, clone number; +, positive control; arrow, targeted product. (**G**) Bioluminescence imaging of C57BL/6J*-Tyr*^*c-2J*^ or *R26*^*LSL-TRL*^ (clone D1) ES cells transduced with increasing multiplicity of infection (MOI) CMV-Cre adenovirus (Ad-Cre). Data are representative of at least three independent replicates. (H) Plot shows flow cytometry measurement of iRFP670 expression in C57BL/6J*-Tyr*^*c-2J*^ or *R26*^*LSL-TRL*^ ES cells transduced with a GMAP-generated tetracycline response element promoter (pTRE):iRFP670-EFS:Cre-2A-GFP lentivirus and treated with doxycycline. Data are representative of at least three independent replicates. **p = 3.71 × 10^−3^; ***p = 6.51 × 10^−4^; ****p = 3.60 × 10^−5^.
