# Supplementary material for: A Modular Assembly Platform for Rapid Generation of DNA Constructs
Source: Sci Rep. 2016 Feb 18;6:16836. doi: 10.1038/srep16836 (PMC4757859; doi:10.1038/srep16836)
Supplement: Supplementary Information [file srep16836-s1.pdf]

SUPPLEMENTARY INFORMATION

A Modular Assembly Platform for Rapid Generation of DNA Constructs

Elliot H. Akama-Garren[1,2,§], Nikhil S. Joshi[1,§], Tuomas Tammela[1], Gregory P. Chang[1], Bethany L. Wagner[1], Da-Yae Lee[1], William M. Rideout III[1], Thales Papagiannakopoulos[1], Wen Xue[3], Tyler Jacks[1,2,4,*]

[1]David H. Koch Institute for Integrative Cancer Research, Massachusetts Institute of Technology, Cambridge, MA 02139

[2]Department of Biology, Massachusetts Institute of Technology, Cambridge, Massachusetts 02142

[3]RNA Therapeutics Institute and Program in Molecular Medicine, University of Massachusetts Medical School, Worcester, MA 01605

[4]Howard Hughes Medical Institute, Massachusetts Institute of Technology, Cambridge, Massachusetts 02139, USA

§These authors contributed equally

*Correspondence and requests for materials should be addressed to T. Jacks (tjacks@mit.edu)

a

Site #1: 5′...GATCAGTGTGAGGGAGTGTAAAGCTGGTTT...3′
Site #2: 5′...CTAACTCGAACGCTAGCTGTGCGATCGTTT...3′
Site #3: 5′...AAACCGCTGTTCCTAGGAATCCCGAGGCCT...3′
Site #4: 5′...GACCCGACATTAGCGCTACAGCTTAAGCGG...3′
Site #5: 5′...AAACGTTGTTGTTTGGGGTTGAATTACTCT...3′

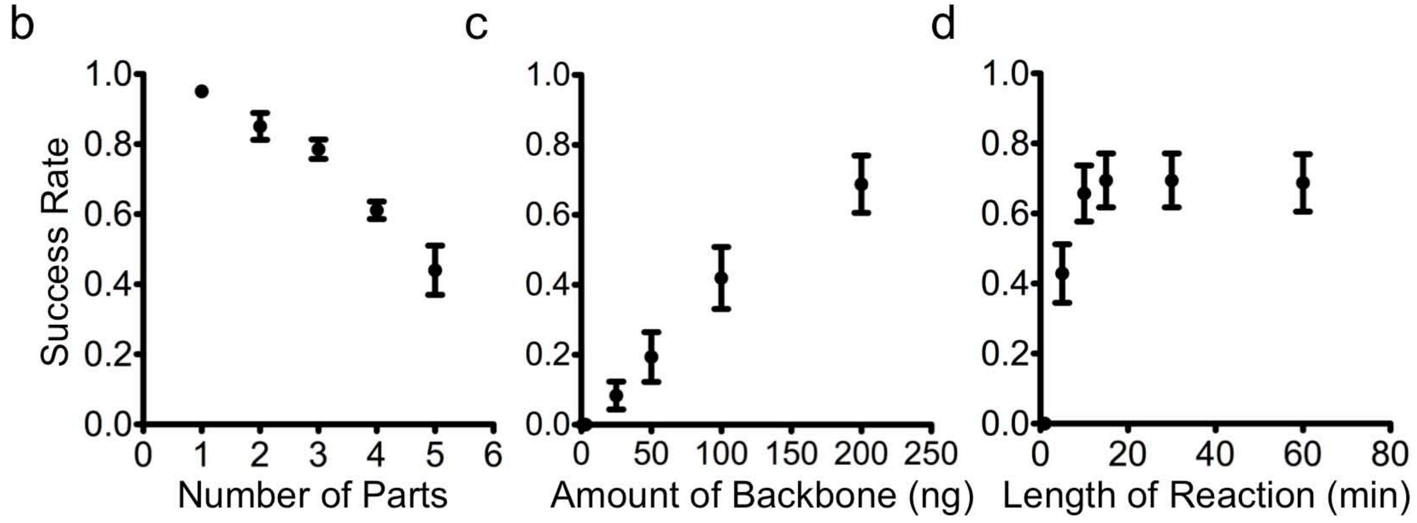

e

<u>GMAP Assembly Protocol</u>

1. Isothermal Assembly (**25 min**)
    1. Add 1 μL of digested backbone and 1 μL of each insert
    2. Bring all reaction volumes to 5 μL using water
    3. Add 15 uL of isothermal master mix to each reaction
    4. Incubate for 20 min at 50°C
2. Transform 2 μL of reaction to 25 μL of competent bacteria (**5 min**)
3. Plate and grow overnight at 37°C (**15 hr**)
4. Pick colonies and inoculate minipreps, grow overnight at 37°C (**12 hr**)
5. Isolate DNA from minipreps (**1 hr**)
6. Screen by digestion with *XmaI, NheI, AvrII, AfeI*, or *AscI* (**30 min**)
7. Inoculate maxiprep with correct colony (**5 min**)

A DNA construct can be assembled from a library of compatible promoters, genes, and backbones in three days. Steps 1-3 can be completed on Day 1, step 4 on Day 2, and steps 5-7 on Day 3.

Supplementary Figure S1. GMAP protocol and timeline

(A) Sequences of five common GMAP sites designed to flank each promoter, gene, or backbone. (B-D) Success rate of GMAP assemblies was calculated as fraction of transformed colonies that digested correctly for varying number of parts in addition to backbone (B), ng of backbone (C), and length of reaction (D). When not varied, four parts were used, 200 ng of backbone was used, and reactions proceeded for 60min. Data are representative of at least twelve transformed colonies. (E) Protocol for GMAP assembly.

a

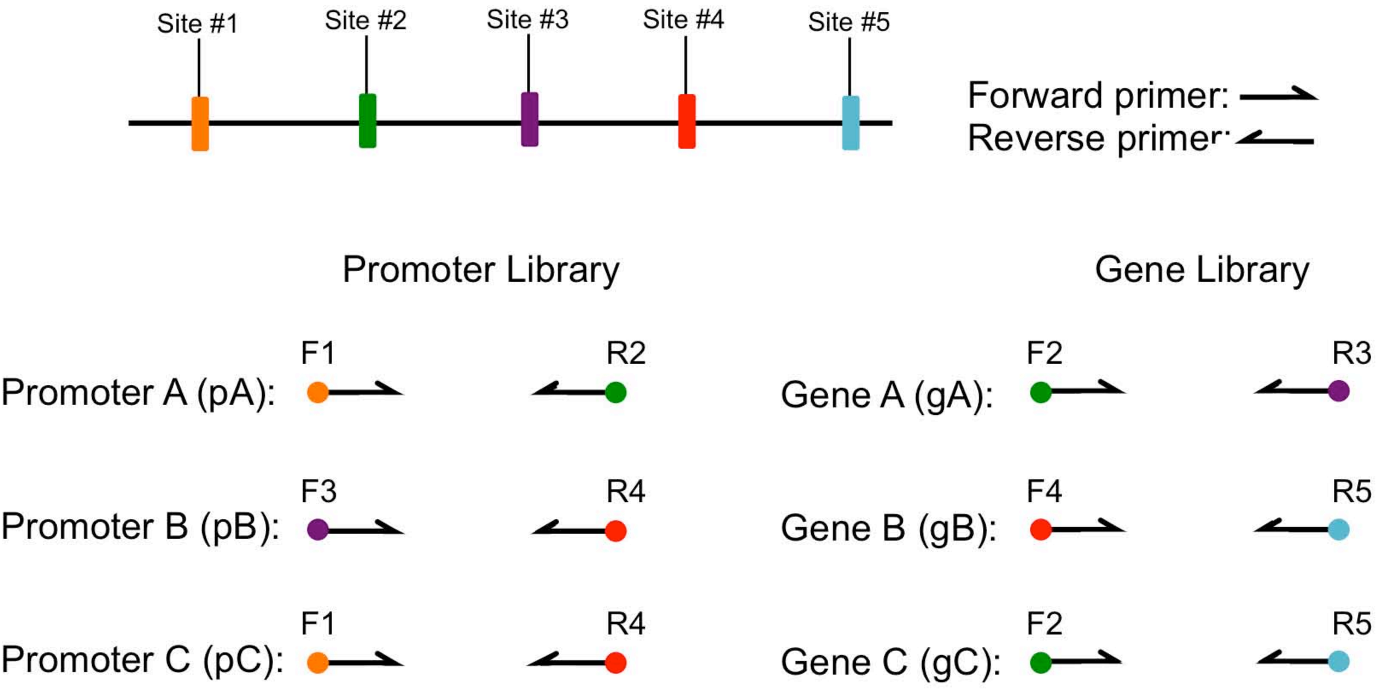

b

<u>GMAP Parts Collection Preparation Protocol</u>

1. For a given **promoter** with known forward primer $(N)_n$ and reverse primer $(N)_n$' , order the following four primers:
   - F1: GATCAGTGTGAGGGAGTGTAAAGCTGGTTT$(N)_n$
   - R2:AAACGATCGCACAGCTAGCGTTCGAGTTAG$(N)_n$'
   - F3: AAACCGCTGTTCCTAGGAATCCCGAGGCCT$(N)_n$
   - R4: CCGCTTAAGCTGTAGCGCTAATGTCGGGTC$(N)_n$'
2. For a given **gene** with known forward primer $(N)_n$* and reverse primer $(N)_n$** , order the following four primers:
   - F2: CTAACTCGAACGCTAGCTGTGCGATCGTTT$(N)_n$*
   - R3:AGGCCTCGGGATTCCTAGGAACAGCGGTTT$(N)_n$**
   - F4: GACCCGACATTAGCGCTACAGCTTAAGCGG$(N)_n$*
   - R5: AGAGTAATTCAACCCCAAACAACAACGTTT$(N)_n$**
3. PCR amplify using scheme shown in (A), as appropriate
4. Gel purify PCR product, adjust to 57nM
5. Add to database and store in collection at -20°C

Supplementary Figure S2. Preparation of promoter and gene collections

(A) PCR scheme for preparation of promoter and gene collections. Sites 1 (orange), 2 (green), 3 (purple), 4 (red), and 5 (turquoise) are represented by rectangles to denote their location in the GMAP assembly scheme, and as circles to denote primer overhangs. (B) Protocol for preparation of promoter and gene collections.

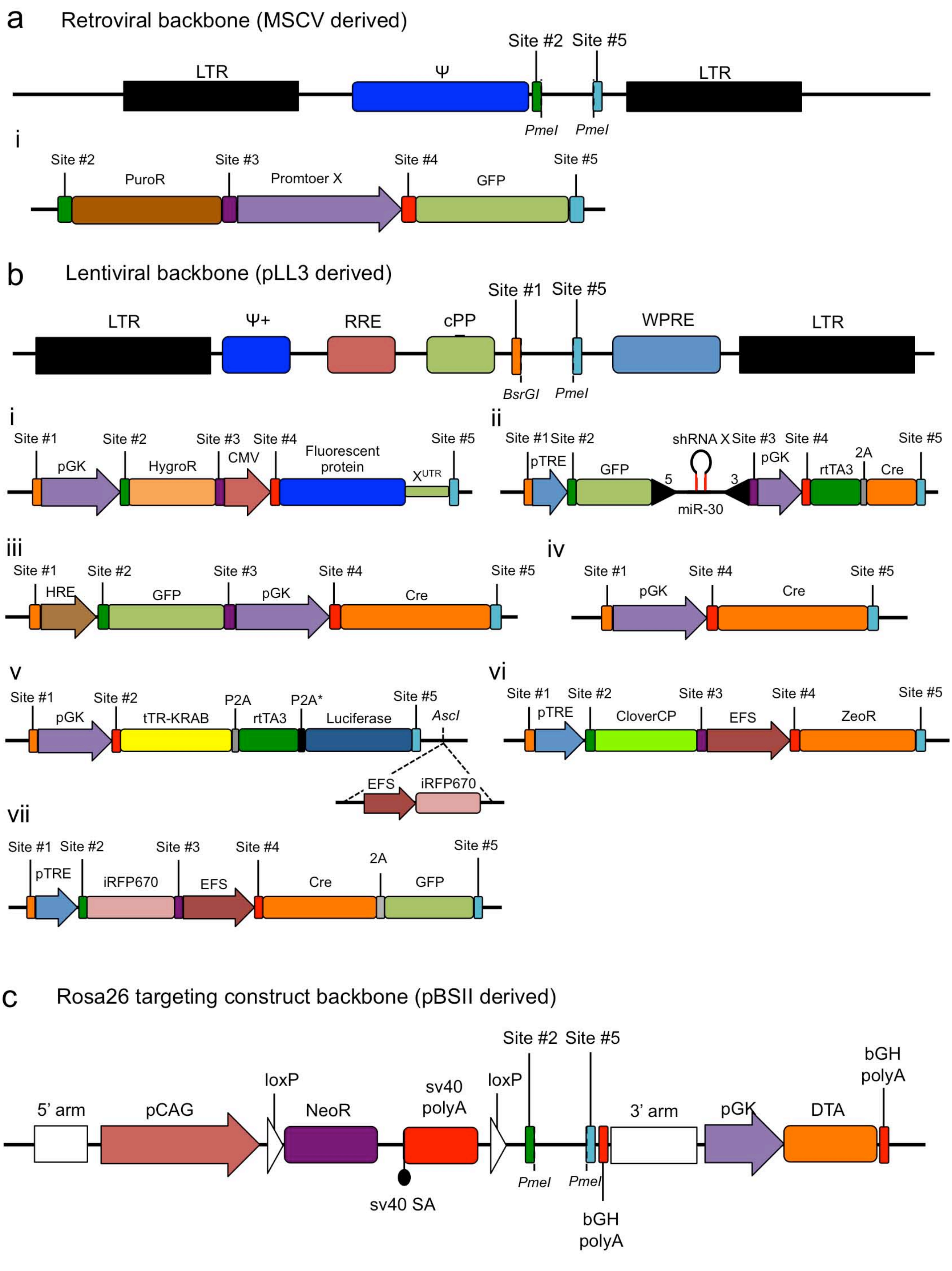
a
Retroviral backbone (MSCV derived)
LTR
Ψ
Site #2
Site #5
LTR
PmeI
PmeI
i
Site #2
Site #3
Site #4
Site #5
PuroR
Promtoer X
GFP
b
Lentiviral backbone (pLL3 derived)
LTR
Ψ+
RRE
cPP
Site #1
Site #5
WPRE
LTR
BsrGI
PmeI
i
Site #1
Site #2
Site #3
Site #4
Site #5
pGK
HygroR
CMV
Fluorescent protein
X^UTR
ii
Site #1
Site #2
shRNA X
Site #3
Site #4
Site #5
pTRE
GFP
5
3
miR-30
pGK
2A
rtTA3
Cre
iii
Site #1
Site #2
Site #3
Site #4
Site #5
HRE
GFP
pGK
Cre
iv
Site #1
Site #4
Site #5
pGK
Cre
v
Site #1
Site #2
P2A
P2A*
Site #5
pGK
tTR-KRAB
rtTA3
Luciferase
AscI
EFS
iRFP670
vi
Site #1
Site #2
Site #3
Site #4
Site #5
pTRE
CloverCP
EFS
ZeoR
vii
Site #1
Site #2
Site #3
Site #4
Site #5
pTRE
iRFP670
EFS
Cre
2A
GFP
c
Rosa26 targeting construct backbone (pBSII derived)
Site #2
Site #5
loxP
loxP
sv40 polyA
bGH polyA
5' arm
pCAG
NeoR
3' arm
pGK
DTA
PmeI
PmeI
sv40 SA
bGH polyA

Supplementary Figure S3. Retroviral, lentiviral, and Rosa26 targeting vector backbones

(A) Map of GMAP-compatible retroviral backbone and retroviral constructs used in Fig. 1d (i). LTR, long terminal repeat; ψ, psi packaging element; PuroR, puromycin resistance; Promoter X represents pGK, CMV, EFS, SV40, CCSP, or UBC; MSCV, murine stem cell virus. (B) Map of GMAP-compatible lentiviral backbone and lentiviral constructs used in Fig. 1e (i), Fig. 1f-h (ii), Fig. 2a (iii, iv), Fig. 2c (v, vi), and Fig. 2h (vii). RRE, HIV Rev response element; cPPT, central polypurine tract; WPRE, woodchuck hepatitis virus posttranscriptional regulatory element; pGK, human phosphoglycerate kinase 1 promoter; HygroR, hygromycin resistance; CMV, cytomegalovirus immediate-early promoter; Fluorescent protein represents mTagBFP2, mKate2, or mKO2; X^{UTR} represents 3'UTR sensor cassette shRNA A, B, or C target sequences; pTRE, tetracycline response element promoter; shRNA X represents shRNA A, B, or C; rtTA3, reverse tetracycline-transactivator; Cre, cre recombinase; HRE, hypoxia response element; tTR-KRAB, tetracycline transrepressor from *Escherichia coli Tn10* fused to the KRAB domain of human Kox1; Luc, luciferase; EFS, elongation factor 1α promoter; ZeoR, zeocin resistance. (C) Map of GMAP-compatible Rosa26 targeting vector backbone. pCAG, cytomegalovirus early enhancer/chicken β actin promoter; NeoR, neomycin resistance; sv40, simian virus 40; SA, splice acceptor; bGH polyA, bovine growth hormone polyadenylation signal; pGK, human phosphoglycerate kinase 1 promoter; DTA, diphtheria toxin fragment A; pBSII, pBluescript II.

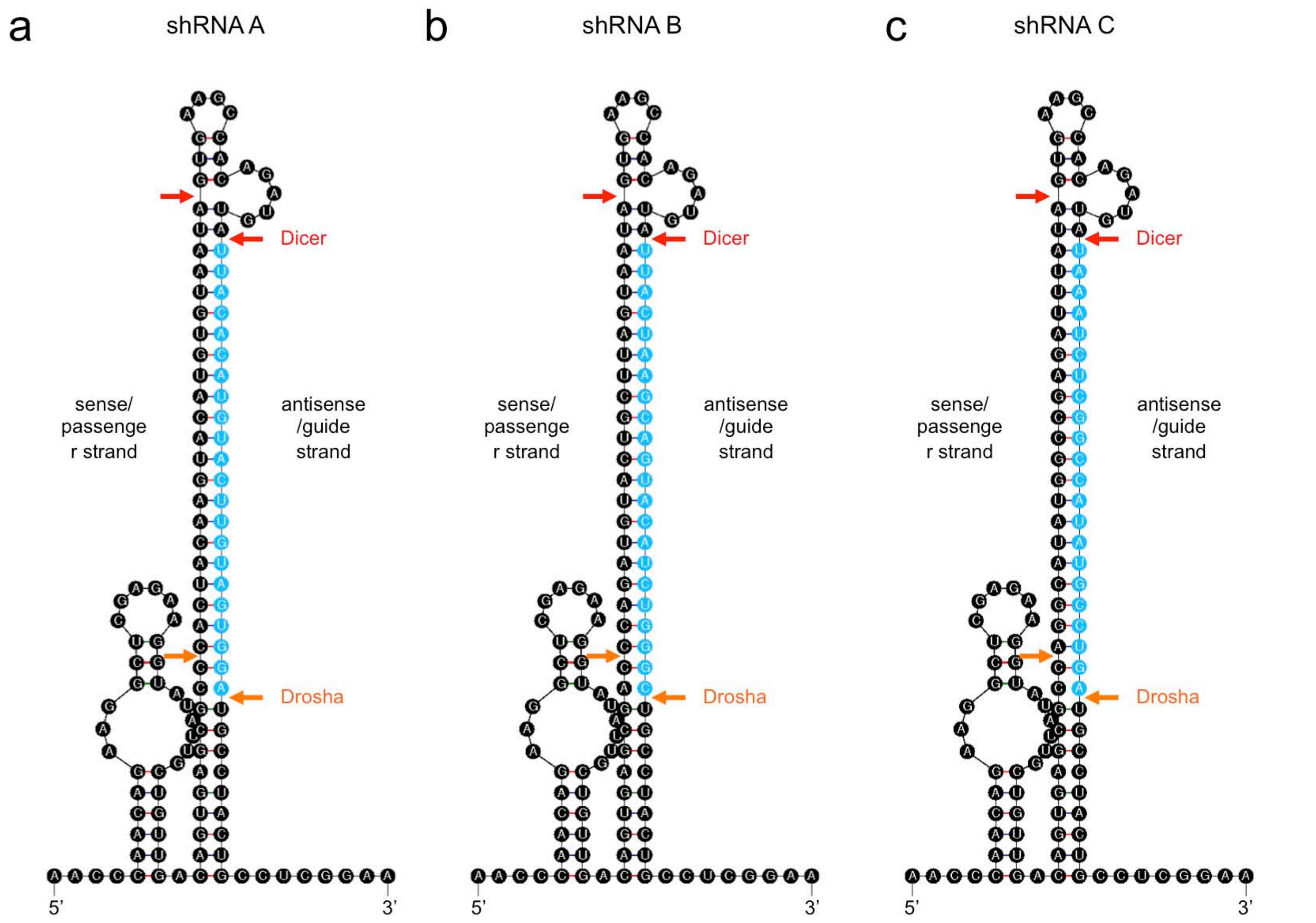

d

3'UTR "Sensor Cassette" mRNA:

	5'flank	Direct Target	3'flank	Site #5	
mTAGBFP2-A^{UTR}:	5'...UGAGUAUACCACCA	UCCACUACAAGUACAUGUGUAA	UAGUCCUUGCAUGG	AAACGUUGUUGUUUGGGGUUGAAUUACUCU	...3'
mKate2-B^{UTR}:	5'...UGAGUAUACCACCA	GCCCAGAUGUACUGCUUAGUAA	UAGUCCUUGCAUGG	AAACGUUGUUGUUUGGGGUUGAAUUACUCU	...3'
mKO2-C^{UTR}:	5'...UGAGUAUACCACCA	UCAGGCAUAUGGCCGAGAUUUA	UAGUCCUUGCAUGG	AAACGUUGUUGUUUGGGGUUGAAUUACUCU	...3'

Supplementary Figure S4. shRNA and sensor cassette sequences

(A-C) Sequences of the miR-30 shRNA cassette highlighting the 22nt guide strand (blue) for shRNA A (A), B (B), and C (C) and its complementary variable region. Canonical cleavage sites for Drosha (orange) and Dicer (red) are annotated[25]. RNA structure prediction was performed using MFold[26]. (D) Sequences of 50 nt 3'UTR sensor cassettes consisting of 5' flanking region, direct target site (green), 3' flanking region, and GMAP site #5 (blue).

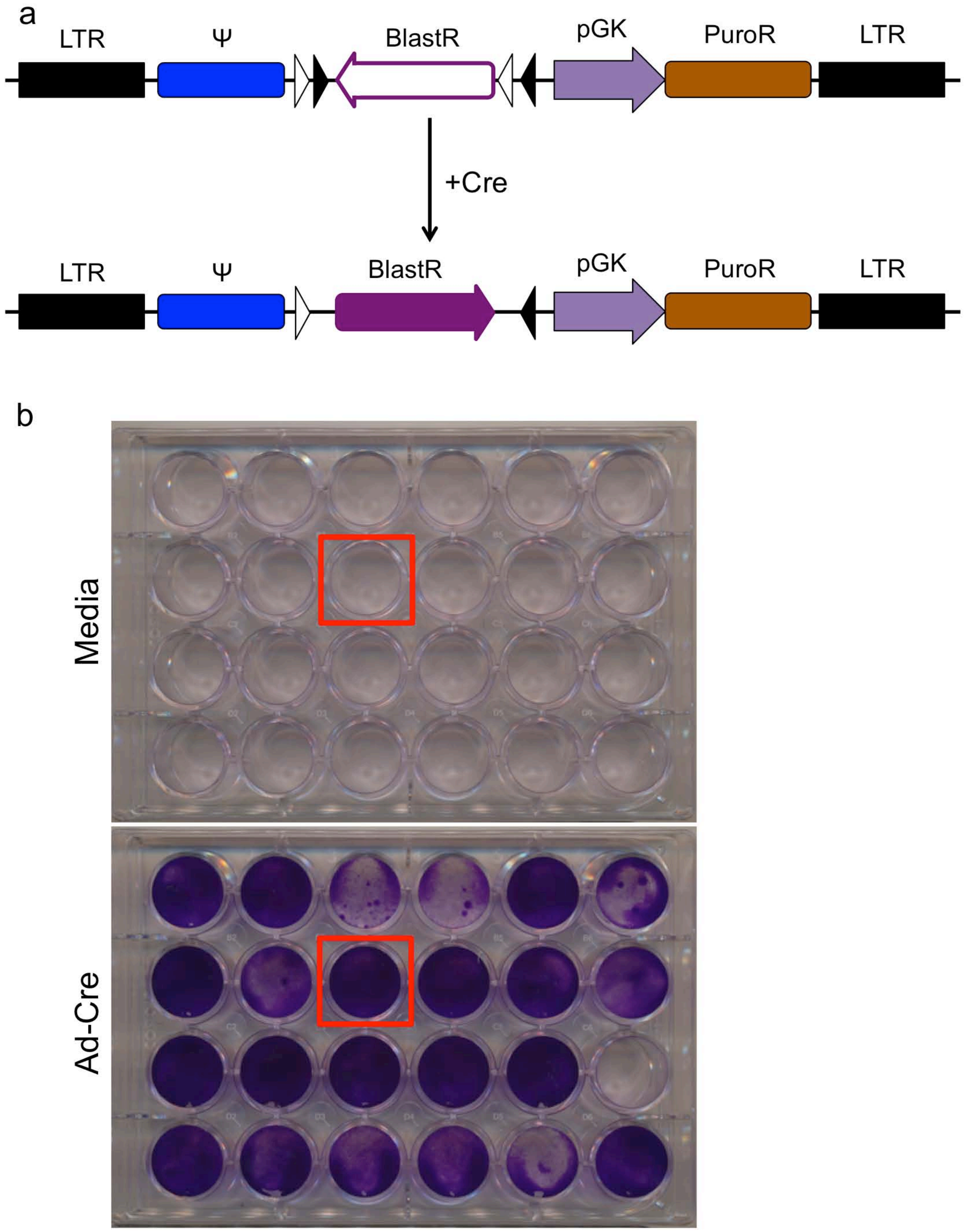
a
LTR
Ψ
BlastR
pGK
PuroR
LTR
+Cre
LTR
Ψ
BlastR
pGK
PuroR
LTR
b
Media
Ad-Cre

Supplementary Figure S5. Establishment of 3TB cell line

(A) Map of FFiBlast MSCV Puro construct. BlastR, blasticidin resitance; arrowheads, two sets of incompatible loxP sites. Top, prior to Cre-mediated inversion; bottom, after Cre-mediated inversion. (B) Crystal violet stain of subclones generated following transduction of murine 3T6 fibroblasts with FFiBlast MSCV Puro. Subclones were transduced with CMV-Cre adenovirus (Ad-Cre) and selected in blasticidin. Red box indicates subclone chosen and expanded for future experiments.

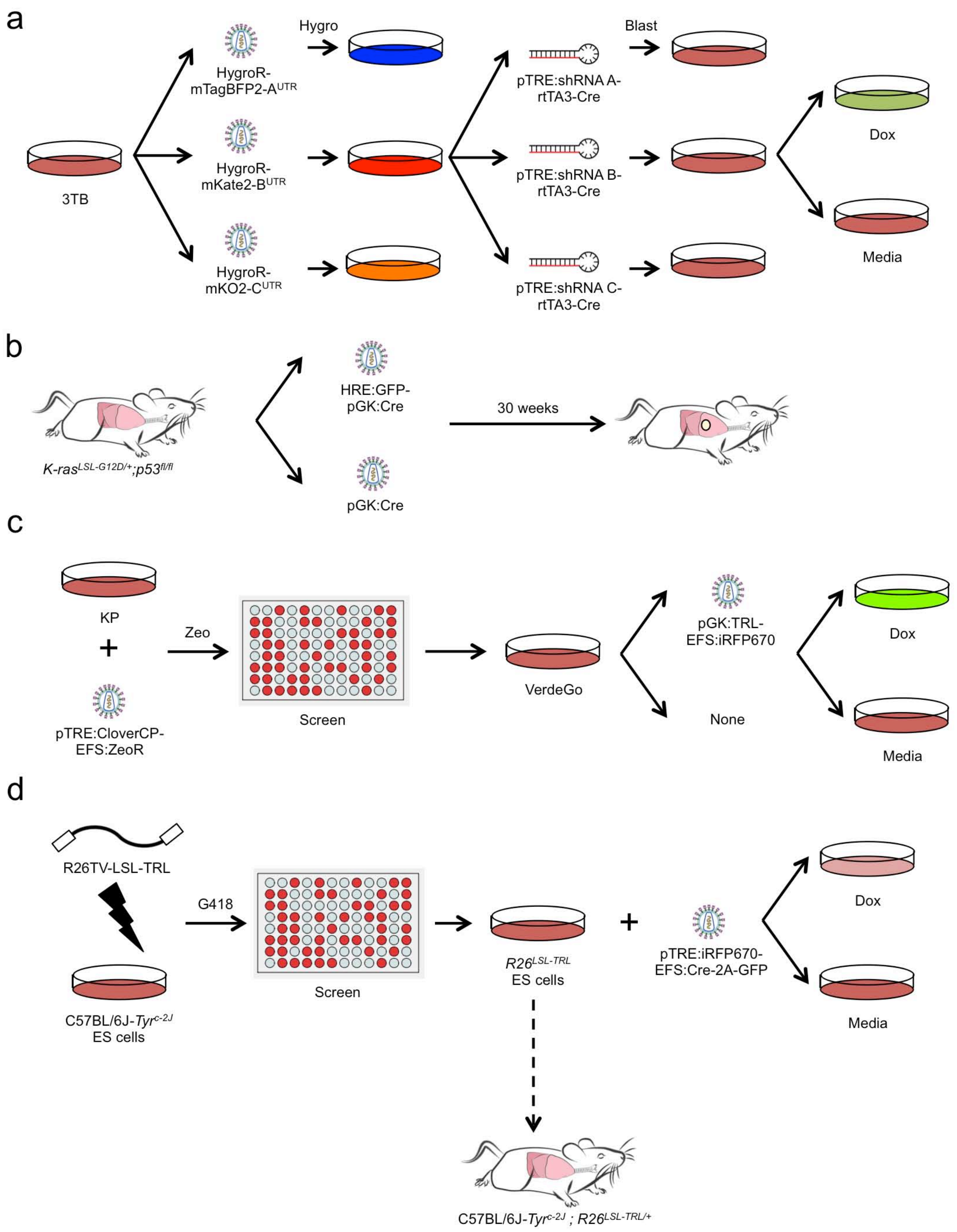
a
Hygro
Blast
HygroR-mTagBFP2-A^UTR
HygroR-mKate2-B^UTR
HygroR-mKO2-C^UTR
3TB
pTRE:shRNA A-rtTA3-Cre
pTRE:shRNA B-rtTA3-Cre
pTRE:shRNA C-rtTA3-Cre
Dox
Media
b
HRE:GFP-pGK:Cre
pGK:Cre
30 weeks
K-ras^LSL-G12D/+;p53^fl/fl
c
KP
Zeo
pTRE:CloverCP-EFS:ZeoR
Screen
VerdeGo
pGK:TRL-EFS:iRFP670
None
Dox
Media
d
R26TV-LSL-TRL
G418
C57BL/6J-Tyr^c-2J ES cells
Screen
R26^LSL-TRL ES cells
pTRE:iRFP670-EFS:Cre-2A-GFP
Dox
Media
C57BL/6J-Tyr^c-2J ; R26^LSL-TRL/+

Supplementary Figure S6. Schematic diagrams of experiments

(A) Schematic diagram of experiment presented in Fig. 1e-h. Lentiviruses expressing mTagBFP2-A^{UTR}, mKate2-B^{UTR}, or mKO2-C^{UTR} were assembled using GMAP and used to transduce 3TB cells. Cells were then selected with hygromycin and transfected with GMAP-assembled inducible hairpin constructs targeting the A, B, or C 3' UTRs. Cells were then selected with blasticidin and treated with doxycycline to assess knockdown efficiency. (B) Schematic diagram of experiment presented in Fig. 2a-b. *K-ras*$^{LSL-G12D/+}$*;p53*$^{fl/fl}$ mice were infected with a GMAP-generated HRE:GFP-pGK:Cre or pGK:Cre lentivirus and after 30 weeks were injected with pimonidazole to visualize areas of hypoxia. (C) Schematic diagram of experiment presented in Fig. 2c-d. KP cells were transduced with a GMAP-generated pTRE:CloverCP-EFS:ZeoR lentivirus and selected with zeocin. Following single cell cloning for a rtTA-dependent and doxycycline-inducible clone, VerdeGo cells were transduced with a GMAP-generated pGK:tTR-KRAB-rtTA3-Luc(TRL)-EFS:iRFP670 lentivirus and treated with doxycycline to validate the TRL cassette. (D) Schematic diagram of experiment presented in Fig. 2e-h. C57BL/6J-*Tyr*$^{c-2J}$ ES cells were electroporated with R26TV-LSL-TRL and selected with G418. ES cells were single cell cloned and screened for proper *Rosa26* homologous recombination by PCR and Southern analyses, then transduced with a GMAP-generated pTRE:iRFP670-EFS:Cre-2A-GFP and treated with doxycycline to functionally test the Cre-dependent TRL knock-in.

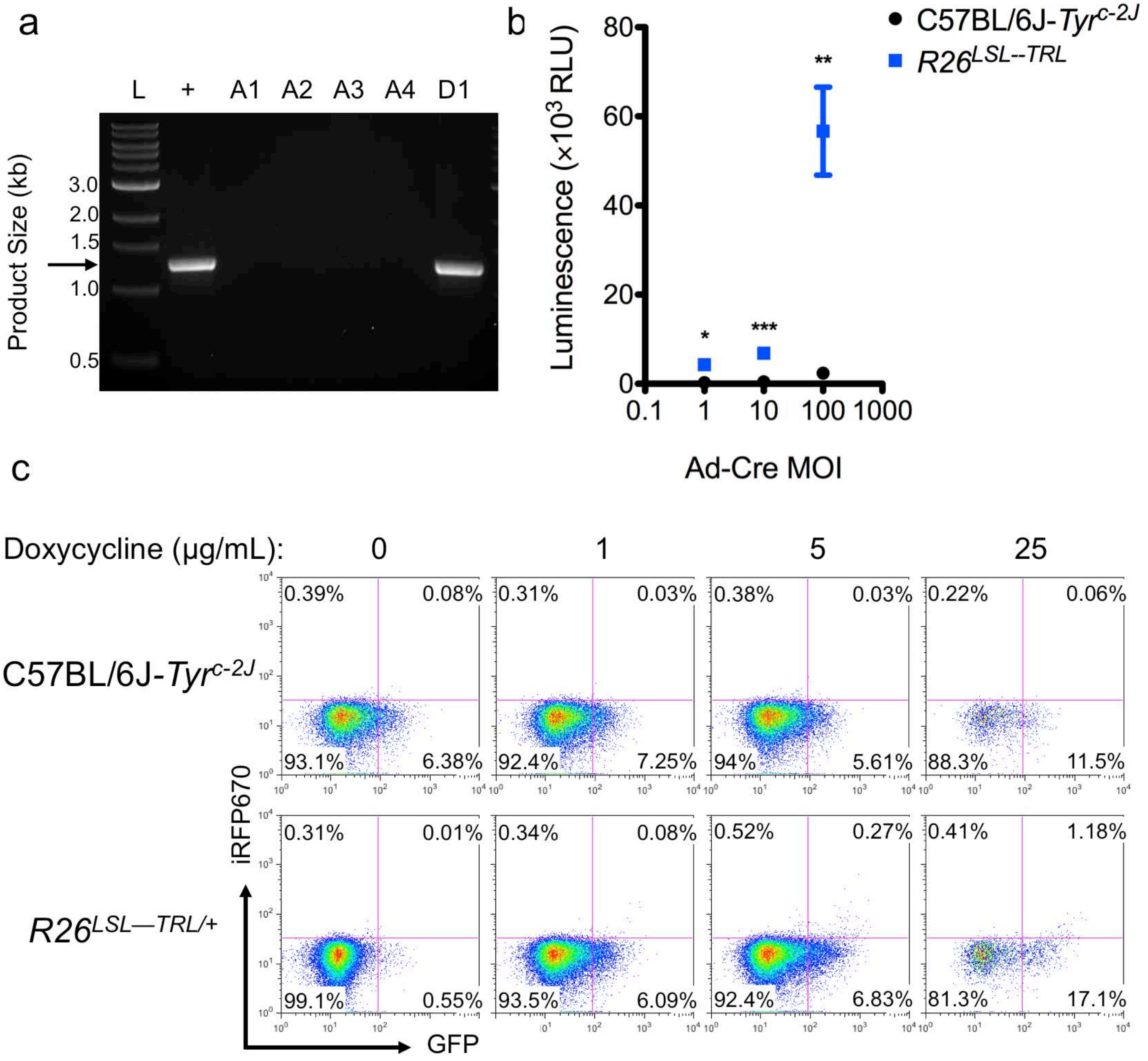
a
L
+
A1
A2
A3
A4
D1
Product Size (kb)
3.0
2.0
1.5
1.0
0.5
b
Luminescence (×10³ RLU)
80
60
40
20
0
0.1
1
10
100
1000
Ad-Cre MOI
C57BL/6J-Tyr^c-2J
R26^LSL--TRL
*
***
**
c
Doxycycline (µg/mL):
0
1
5
25
C57BL/6J-Tyr^c-2J
R26^LSL—TRL/+
0.39%
0.08%
93.1%
6.38%
0.31%
0.03%
92.4%
7.25%
0.38%
0.03%
94%
5.61%
0.22%
0.06%
88.3%
11.5%
0.31%
0.01%
99.1%
0.55%
0.34%
0.08%
93.5%
6.09%
0.52%
0.27%
92.4%
6.83%
0.41%
1.18%
81.3%
17.1%
iRFP670
GFP

Supplementary Figure S7. Screening and functional validation of R26$^{LSL\text{-}TRL}$ *ES cells*

(A) PCR screen of ES cell clones. ES cell subclones following R26TV-LSL-TRL electroporation were generated and PCRs were performed on isolated genomic DNA using R26For and R26Rev (L, 1 kb ladder; A1-D1, clone number; +, positive control; arrow, 1302 bp targeted product; kb, kilobases). (B) Bioluminescence measurements from cell lysates of C57BL/6J-*Tyr*$^{c\text{-}2J}$ or *R26*$^{LSL\text{-}TRL}$ (clone D1) ES cells transduced with increasing multiplicity of infection (MOI) CMV-Cre adenovirus (Ad-Cre). Data are representative of at least three independent replicates. RLU, relative light units; *, $p=1.48\times10^{-3}$; **, $p=1.08\times10^{-3}$; ***, $p=1.21\times10^{-4}$. (C) Representative FACS plots show iRFP670 and GFP expression from C57BL/6J-*Tyr*$^{c\text{-}2J}$ or *R26*$^{LSL\text{-}TRL}$ ES cells transduced with pTRE:iRFP670-EFS:Cre-2A-GFP lentivirus and treated with doxycycline. Data are representative of at least three independent replicates.
